# *Fusarium* Species Infecting Greenhouse-Grown Cannabis (*Cannabis sativa*) Plants Show Potential for Mycotoxin Production in Inoculated Inflorescences and from Natural Inoculum Sources

**DOI:** 10.3390/jof11070528

**Published:** 2025-07-16

**Authors:** Zamir K. Punja, Sheryl A. Tittlemier, Sean Walkowiak

**Affiliations:** 1 Department of Biological Sciences, Simon Fraser University, Burnaby, BC V5A 1S6, Canada; 2 Grain Research Laboratory, Canadian Grain Commission, Winnipeg, MB R3C 3G8, Canadasean.walkowiak@grainscanada.gc.ca (S.W.)

**Keywords:** *Fusarium avenaceum*, *Fusarium graminearum*, bud rot, mycotoxins, post-harvest

## Abstract

Several species of *Fusarium* are reported to infect inflorescences of high-THC-containing cannabis (*Cannabis sativa* L.) plants grown in greenhouses in Canada. These include *F. graminearum, F. sporotrichiodes*, *F. proliferatum*, and, to a lesser extent, *F. oxysporum* and *F. solani*. The greatest concern surrounding the infection of cannabis by these *Fusarium* species, which cause symptoms of bud rot, is the potential for the accumulation of mycotoxins that may go undetected. In the present study, both naturally infected and artificially infected inflorescence tissues were tested for the presence of fungal-derived toxins using HPLC-MS/MS analysis. Naturally infected cannabis tissues were confirmed to be infected by both *F. avenaceum* and *F. graminearum* using PCR. Pure cultures of these two species and *F. sporotrichiodes* were inoculated onto detached inflorescences of two cannabis genotypes, and after 7 days, they were dried and assayed for mycotoxin presence. In these assays, all *Fusarium* species grew prolifically over the tissue surface. Tissues infected by *F. graminearum* contained 3-acetyl DON, DON, and zearalenone in the ranges of 0.13–0.40, 1.18–1.91, and 31.8 to 56.2 μg/g, respectively, depending on the cannabis genotype. In *F. sporotrichiodes*-infected samples, HT2 and T2 mycotoxins were present at 13.9 and 10.9 μg/g in one genotype and were lower in the other. In *F. avenaceum*-inoculated tissues, the mycotoxins enniatin A, enniatin A1, enniatin B, and enniatin B1 were produced at varying concentrations, depending on the isolate and cannabis genotype. Unexpectedly, these tissues also contained detectable levels of 3-acetyl DON, DON, and zearalenone, which was attributed to apre-existing natural infection by *F. graminearum* that was confirmed by RT-qPCR. Beauvericin was detected in tissues infected by *F. avenaceum* and *F. sporotrichiodes*, but not by *F. graminearum*. Naturally infected, dried inflorescences from which *F. avenaceum* was recovered contained beauvericin, enniatin A1, enniatin B, and enniatin B1 as expected. Uninoculated cannabis inflorescences were free of mycotoxins except for culmorin at 0.348 μg/g, reflecting pre-existing infection by *F. graminearum*. The mycotoxin levels were markedly different between the two cannabis genotypes, despite comparable mycelial colonization. Tall fescue plants growing in the vicinity of the greenhouse were shown to harbor *F. avenaceum* and *F. graminearum*, suggesting a likely external source of inoculum. Isolates of both species from tall fescue produced mycotoxins when inoculated onto cannabis inflorescences. These findings demonstrate that infection by *F. graminearum* and *F. avenaceum*, either from artificial inoculation or natural inoculum originating from tall fescue plants, can lead to mycotoxin accumulation in cannabis inflorescences. However, extensive mycelial colonization following prolonged incubation of infected tissues under high humidity conditions is required. Inoculations with *Penicillium citrinum* and *Aspergillus ochraceus* under these conditions produced no detectable mycotoxins. The mycotoxins alternariol and tentoxin were detected in several inflorescence samples, likely as a result of natural infection by *Alternaria* spp. *Fusarium avenaceum* is reported to infect cannabis inflorescences for the first time and produces mycotoxins in diseased tissues.

## 1. Introduction

The commercial production of high-THC-containing plants of cannabis (*Cannabis sativa* L.) in Canada following legalization in 2018 for recreational and medicinal uses has been steadily increasing. Production occurs mostly in environmentally controlled indoor rooms and in greenhouses, although a small proportion of the production, approximately 10%, occurs outdoors. One of the major challenges to the large-scale production of cannabis plants is the potential exposure to infection by pathogens, which may originate from various sources. These sources include infected propagation materials, contaminated seeds, or inoculum from adjacent fields in which the pathogens may be infecting unrelated crops, which subsequently provide a source of inoculum for cannabis plants [1]. There is previously published evidence that a large number of fungal pathogens with wide host ranges are able to spread and infect cannabis plants under the appropriate environmental conditions [1,2].

There are currently no regulatory restrictions to prevent the spread of pathogens within or between cannabis crops or across geographical regions where cannabis production is permitted. Hence, the unregulated movement of plant materials from one region to another has been shown to result in the spread of a number of pathogens contained in that material [1]. Regulations are imposed on the occurrence of specific fungal pathogens that have the potential to produce mycotoxins within the cannabis inflorescences that are harvested and marketed for sale [3]. These regulations specify the tolerance levels for certain species of *Aspergillus* and *Penicillium* species and their associated mycotoxins. In some jurisdictions, these tolerance levels may be zero. There is currently little published information on the extent to which these mycotoxins may be present and the corresponding levels in different jurisdictions [3,4].

Recently, reports of infection by a range of mycotoxin-producing species of *Fusarium* on both greenhouse-grown cannabis plants as well as on field-grown hemp plants have raised concerns over the potential for mycotoxin production within the inflorescences, and hence their potential to cause harm to humans and animals exposed to these contaminating species [5,6,7,8]. In hemp plants grown outdoors, several mycotoxins produced by *Fusarium graminearum* have been reported to be present in inflorescences [7]. These plants were generally exposed to prolonged periods of wet weather outdoors that provided conducive conditions for growth of these pathogens. The sources of inoculum were identified to be adjacent wheat or cereal fields harboring these pathogens [6,8]. In greenhouse-grown cannabis plants in Canada, the occurrence of potential mycotoxin-producing species of *Fusarium* that include *F. graminearum* and *F. sporotrichiodes* has been reported [1,2,9], but there are no previous reports of the occurrence of associated mycotoxins in the infected inflorescences. In addition, the potential sources of inoculum of *Fusarium* species that are able to infect these tissues and the environmental conditions favoring disease development are unknown.

**Figure 1:**
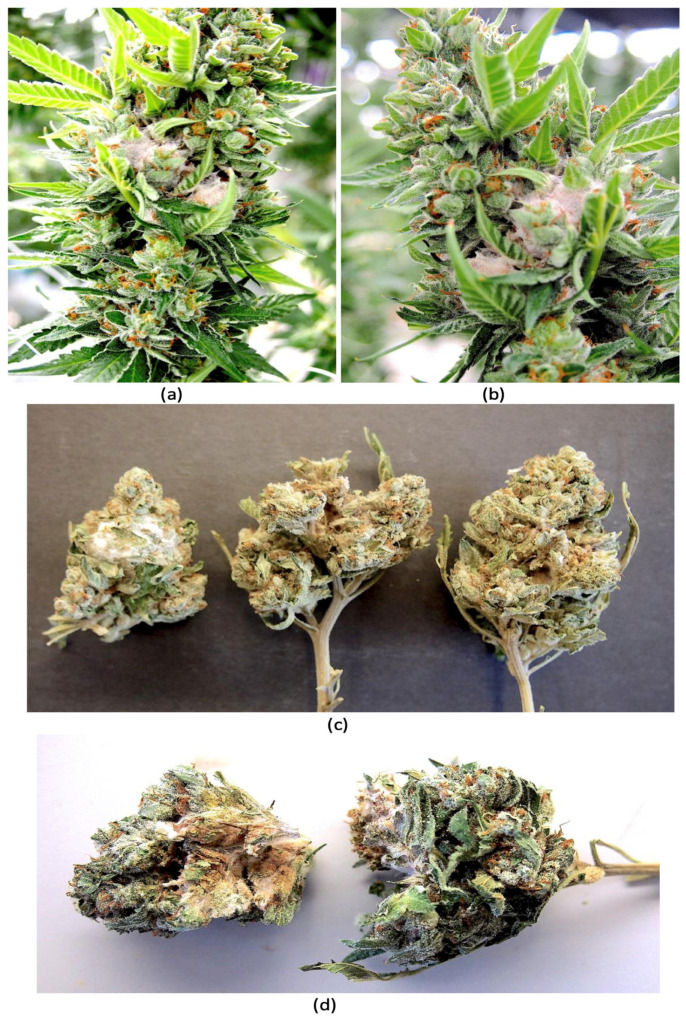
Observations of mycelium of *Fusarium* spp. growing on cannabis inflorescences. (**a**,**b**) Pinkish-white mycelial growth can be seen on inflorescences on plants in the greenhouse from natural infection. (**c**) Mycelial growth is evident on dried inflorescences from the same plants. (**d**) Pinkish-white mycelial growth can be seen inside and on the surface of dried buds. The presence of *Fusarium* spp. was confirmed by isolation in pure culture and molecular identification.

In 2024, cannabis plants showing pinkish-white mycelial growth on the inflorescences were observed in the greenhouse environment of a producer in British Columbia (Figure 1a,b). Samples of dried cannabis inflorescences after the drying process was completed and before packaging had occurred also showed evidence of infection. These samples showed pinkish-white mycelial growth indicative of the presence of a *Fusarium* sp. on the exterior of the sample as well as internally (Figure 1c,d). The objectives of this study were to (i) confirm the species of *Fusarium* that were present on these inflorescences and to determine if the tissues contained any mycotoxins; (ii) to conduct artificial inoculation studies to demonstrate pathogenicity of the isolates recovered and to determine if there were any mycotoxins produced; and (iii) to investigate possible sources of inoculum that could explain the occurrence of infection on greenhouse-grown inflorescences.

## 2. Materials and Methods

*Plant and fungal sources.* During July 2024, visible symptoms of infection of the inflorescences of cannabis plants of genotype AP were observed in a licensed commercial greenhouse facility situated in the Fraser Valley region of British Columbia. The affected inflorescences developed a whitish-pink mycelium (Figure 1a,b) that was visibly different from that commonly caused by *Botrytis cinerea*, the gray mold pathogen that causes bud rot [5,6]. Small tissue pieces measuring 0.5 mm^2^ were dissected from the affected inflorescences and surface-sterilized for 90 s in a 10% solution of household bleach (containing 5.25% NaOCl) followed by a 30 s dip in 70% EtOH and then rinsed thrice in sterile distilled water. The pieces were blotted dry and plated onto potato dextrose agar containing 130 mg/L of streptomycin sulfate (PDA+S) and incubated under ambient laboratory conditions (temperature range of 21–23 °C and 10–12 h/day fluorescent lighting) for 5–7 days. Emerging colonies were sub-cultured onto fresh media and kept for further identification using morphological and molecular criteria.

**Figure 2:**
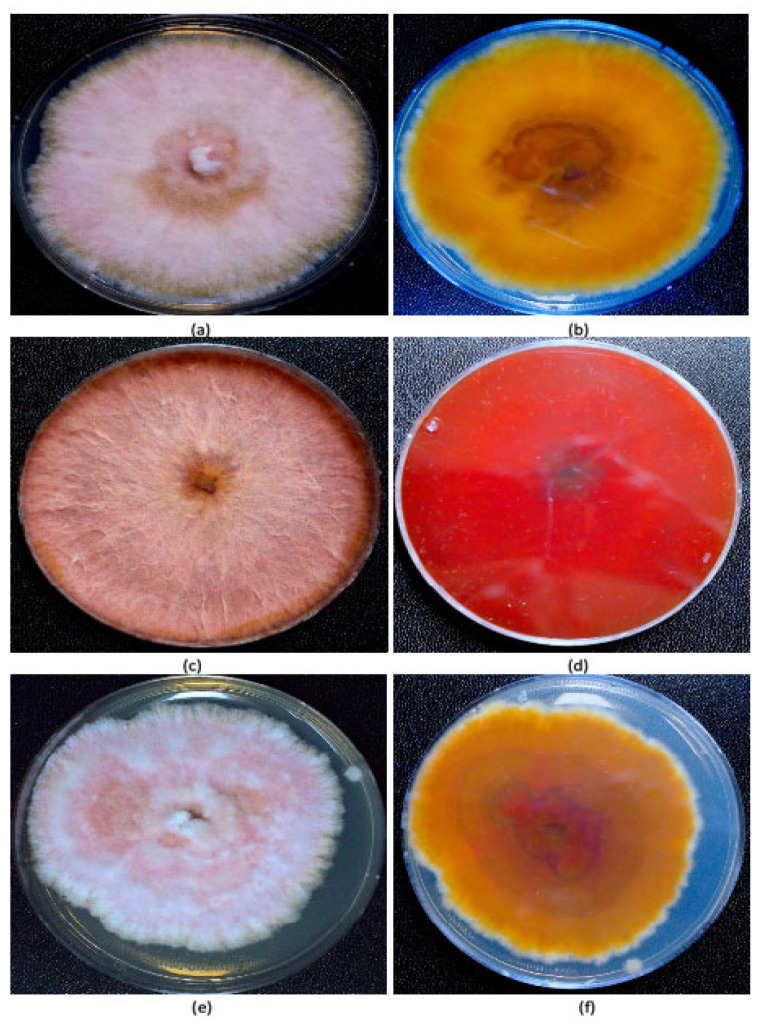
Colony morphology of two *Fusarium* spp. growing on potato dextrose agar for 2 weeks. (**a**,**b**) Top view and bottom view, respectively, of *Fusarium avenaceum* isolated from cannabis inflorescences shown in Figure 1a,b. (**c**,**d**) Top view and bottom view, respectively, of *Fusarium graminearum* isolated from cannabis inflorescences shown in Figure 1a,b. (**e**,**f**) Top view and bottom view, respectively, of *Fusarium avenaceum* isolated from tall fescue plants.

In August of 2024, samples of cannabis inflorescences of genotype AD from the same greenhouse facility were observed to contain a whitish-pink mycelium on the surface of the tissues as well as internally, causing visible decay (Figure 1c,d). These samples had been harvested and dried following commercial practices and were being sorted after trimming and before packaging. The tissues were subjected to the isolation procedure described above. Additional sampling was conducted during August of 2024 of the florets (inflorescences) of tall fescue (*Festuca arundinacea* Schreb.) plants that were growing outside the greenhouse in which diseased cannabis inflorescences were observed. These plants were situated at a distance of approximately 30 m away from the greenhouse (Figure 2a,b). Samples of inflorescences and leaves were obtained from these plants at random and surface-sterilized as above and plated onto PDA+S and select fungal colonies were transferred to fresh medium after 5–7 days. The weather conditions during sampling were overcast skies with a light drizzle and a temperature of 18 °C.

*Molecular identification.* A number of fungal colonies that were consistently recovered on Petri dishes from the tissue samples of cannabis and tall fescue inflorescences were sub-cultured and examined for colony color and growth pattern. Most colonies produced a reddish-brown pigment and resembled *Fusarium* spp. Others were darkly pigmented and resembled *Alternaria* species. Six representative isolates of *Fusarium* spp. as well as three unidentified cultures from diseased inflorescences or leaves were transferred from hyphal-tipped colonies and sent to the University of Guelph Laboratory Services, Agriculture and Food Laboratory, Guelph, ON (www.guelphlabservices.com, accessed on 4 August 2024) for identification to the species level by PCR using the primers ITS1–ITS4 (ITS1-F CTTGGTCATTTAGAGGAAGTAA and ITS4 TCCTCCGCTTATTGATATGC). The elongation factor 1α (EF-1α) primer set EF-1 (5′ ATG GGT AAG GAG GAC AAG AC 3′) and EF-2 (5′ GGA GGT ACC AGT GAT CAT GTT 3′) [2] was also used for further speciation of *Fusarium* spp. as follows. Cultures of representative isolates from different tissue sources were grown in potato dextrose broth at room temperature for 7 days and DNA was extracted from harvested mycelium using the QIAGEN DNeasy Plant Mini Kit (QIAGEN, Montreal, QC, Canada). Aliquots of 1 μL containing 5–20 ng DNA were used for PCR in a 25 μL reaction volume consisting of 2.5 μL 10x buffer (containing 15 mM MgCl_2_), 0.5 μL 10 mM dNTP, 0.25 μL Taq DNA Polymerase (Qiagen, Montreal, QC, Canada), 0.25 μL 10 mM forward and reverse primers, as well as 20.25 μL DNAse- and RNAse-free water (Invitrogen, Waltham, MA, USA). All PCR amplifications were performed in a MyCycler thermocycler (BioRad, Hercules, CA, USA) with the following program: 3 min at 94 °C; 30 s at 94 °C, 30 s at 60 °C, 3 min at 72 °C (35 cycles); and 7 min at 72 °C. PCR products were separated on 1% agarose gels and bands of the expected size (700 bp) were purified with the QIAquick Gel Extraction Kit (QIAGEN, Montreal, QC, Canada) and sent to Eurofins Genomics (Eurofins MWG Operon LLC 2016, Louisville, KY, USA) for sequencing. The resulting sequences were compared to the corresponding ITS1-5.8S-ITS4 or EF-1α sequences from the National Center for Biotechnology Information (NCBI) GenBank database (www.ncbi.nlm.nih.gov, accessed on 4 September 2024). Multiple sequence alignment of the respective isolates was performed using the CLUSTAL W program (http://www.genome.jp/tools/clustalw, accessed on 10 September 2024). Species identities were also confirmed with species-specific DNA markers following the methods as described by Bamforth et al. [10] for both DNA samples isolated from pure fungal cultures and from naturally and artificially infected plant tissues.

*Pathogenicity tests*. Following the identification of the fungal isolates to the species level, five *Fusarium* isolates representing two species were used for pathogenicity tests. The five isolates included three *F. avenaceum* isolates—two from cannabis inflorescences (fresh and dried, FB1 and FB2) and the other isolate from tall fescue inflorescences (FB3)—one isolate of *F. graminearum* from tall fescue plants (FG), and a *F. sporotrichiodes* isolate (FS) from a diseased cannabis inflorescence obtained in 2021 [2,9]. The five isolates were grown on PDA+S for 2 weeks and mycelial plugs (8 mm diameter) were placed mycelial side down onto freshly harvested detached cannabis inflorescences of two cannabis genotypes—AK and AD. The characteristic features of these two genotypes are described in Supplementary Figure S1. Three mycelial plugs were placed equidistant from one another on each inflorescence sample. The inflorescences were harvested from 7-week-old flowering plants in the same greenhouse facility. They were chosen at random but ensuring there were no visible signs of any fungal infection. They were placed inside plastic bags and transported to the laboratory and stored at 4 °C overnight. Two inflorescences were each placed inside plastic containers (25 cm × 12 cm) lined with wet paper towels. Following inoculation, the tissues were misted thrice with sterile distilled water from a hand-held spray bottle and the lids were closed. There were two containers for each of the five isolates (*n* = 4 per isolate) and they were incubated under ambient laboratory conditions. Control inflorescences were similarly misted but not inoculated. In addition to the five *Fusarium* isolates, an isolate each of *Penicillium citrinum* and *Aspergillus ochraceous* was also inoculated onto a duplicate set of inflorescences and placed under the same conditions. These cultures were obtained from cannabis inflorescence tissues in a previous study [11]. All containers were examined at 4 and 7 days after inoculation, and each inoculated sample was photographed to show the extent of mycelial development of the respective fungi. The experiment was repeated a second time in September 2024 using a different set of inflorescences of the same two genotypes harvested from 7-week-old flowering plants and inoculated as described previously. Thus, each fungal isolate was tested for pathogenicity in 8 replications on cannabis inflorescences of 2 genotypes from 2 independent trials.

*Mycotoxin analysis*. After 7 days of incubation, all inflorescence samples were gently transferred to large trays and placed inside a forced air incubator set at 35 °C for 5 days or until all moisture had been removed and the samples felt dry to the touch. They were then transferred to Ziploc bags and sealed and stored inside a dark cabinet for 3 months. Prior to mycotoxin analysis, one inflorescence sample from each of the repeated experiments was combined to represent the specific fungal species inoculated. Control samples were combined in the same manner. The samples were crumbled by hand and tissue pieces were crushed using a mortar with a pestle until a comminuted mixture of small fragments was obtained. They were transferred into a 50 mL Falcon tube and stored for 2 weeks in darkness. The contents were thoroughly shaken by hand and a 5 g subsample was transferred to a small screw-cap vial and prepared for analysis for mycotoxins as described by Brown et al. [12]. A sub-sample of 0.1 g of the ground dried cannabis tissue was transferred to a 30 mL glass test tube to which 1 mL of acetonitrile was added. The tubes were capped and rolled vertically by hand for 30 s to completely coat and submerge the tissues with solvent, followed by sonication for 5 min. The tubes were rolled vertically by hand once again for 30 s, and the solvent was injected directly into an LC vial and subjected to the HPLC-MS/MS protocol described below.

A method based on high-performance liquid chromatography (HPLC) with tandem mass spectrometry (HPLC-MS/MS) was used for analyte (mycotoxin) determination [13]. The HPLC-MS/MS method covered 28 analytes, and the limits of quantitation are shown in Table 1. Stable isotope labeled ^13^C internal standards were added to the diluted sample extract prior to HPLC-MS/MS analysis to mitigate the impact of matrix effects. Quality control samples were processed and analyzed alongside the 12 cannabis samples to monitor analytical method performance.

Analysis was performed on an AB Sciex 5500 TQ tandem mass spectrometer (AB Sciex, Concord, ON, Canada) coupled to a Waters Acquity I Class ultra-high-pressure liquid chromatography system consisting of an Acquity binary solvent manager, an Acquity FTN sample manager, and an Acquity column manager fitted with a Phenomenex Kinetex (Phenomenex, Torrance, CA, USA) 2.6 μm XB-C18 (50 × 2.1 mm) column held at 40 °C. A linear gradient HPLC program using 95.5% 5 mM ammonium acetate in water/0.5% acetic acid (*v*/*v*) and 95.5% 5 mM ammonium acetate in methanol with 0.5% acetic acid (*v*/*v*) as mobile phases A and B, respectively, separated the mycotoxin analytes shown in Table 1. The initial mobile phase was 98% A, changing to 30% A at 15 min and 2% A at 15.7 min. The mobile phase was changed back to the initial 98% A at 18.2 min and held there until the end of the run at 20 min. Mass spectrometric analysis was performed using electrospray ionization with polarity switching. The following mass spectrometer parameters were used: ion spray voltage of ±4500 V (depending on analyte), source temperature of 550 °C, curtain gas (N_2_) at 25 psi, ion source gases (N_2_) at 60 and 70 psi, and collision gas (N_2_) at 9 psi. The transitions monitored and used to identify and quantify each of the mycotoxin analytes are described by Tittlemier et al. [13].

Mycotoxin analytes were quantified using a calibration curve constructed from seven external standards. Peak areas from quantitation transitions were normalized to the peak area of ^13^C-labeled internal standard during data analysis in order to counter matrix effects on quantitation prior to the interpolation of analyte concentration from the calibration curve. Isotope-labeled structural analogs were not commercially available for AOH, AME, ALT, TENT, BEAU, ENN A, ENN A1, ENN B, and ENN B1; therefore, peak areas from their quantitation transitions were normalized to ^13^C_34_-FB_1_. Analyte recoveries were calculated from pre-extraction fortified blanks analyzed with each sample batch. Mycotoxin concentrations in samples were corrected for recovery using the batch-specific recoveries determined for the pre-extraction fortified blanks. Analytes were considered to be identified and were quantified in samples when their retention time was within 0.1 min of the mean retention time in external standards used to construct the calibration curve, the ratio of qualifier to quantitation transition was within ±30% of the mean ratio in external standards used to construct the calibration curve, and the peak area signal-to-noise ratio was at least 10:1. Limits of quantitation were determined as the concentration in sample that produced a chromatographic peak with a signal-to-noise ratio of 10:1.

## 3. Results

**Figure 3:**
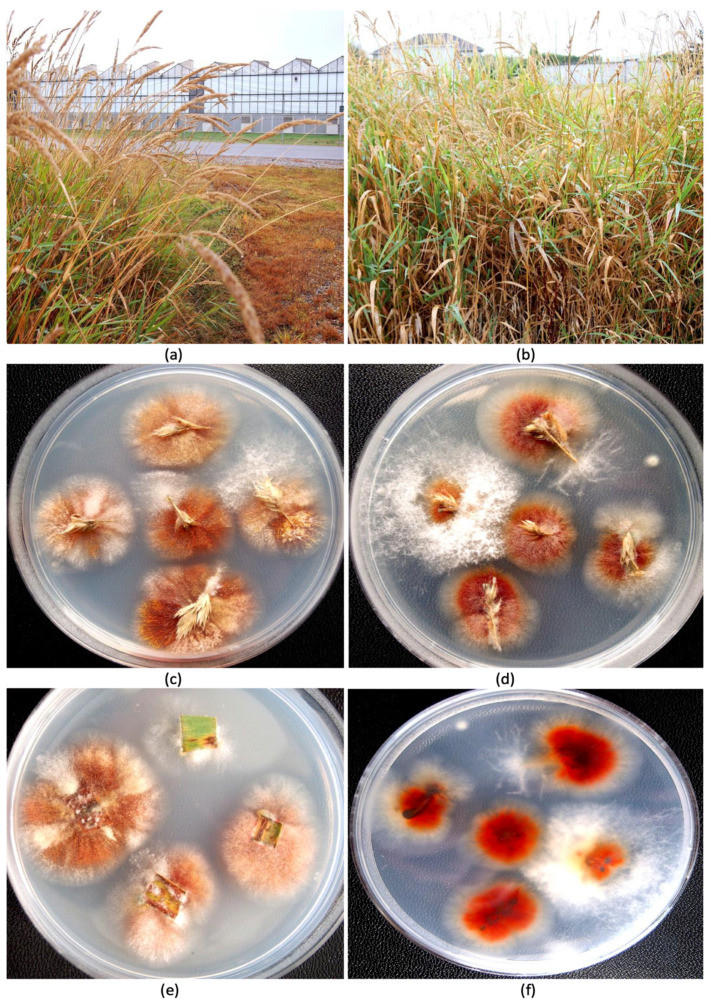
Recovery of fungal species from tall fescue plants growing adjacent to a cannabis greenhouse. (**a**,**b**) Tall fescue plants in the foreground with the cannabis greenhouse in the background. Photos were taken in August 2024 when the plants had developed abundant flowers. (**c**–**f**) Colonies of fungi recovered from inflorescence tissues plated onto potato dextrose agar. A high frequency of red-pigmented colonies was observed, which were identified as species of *Epicoccum* and *Fusarium*.

**Figure 4:**
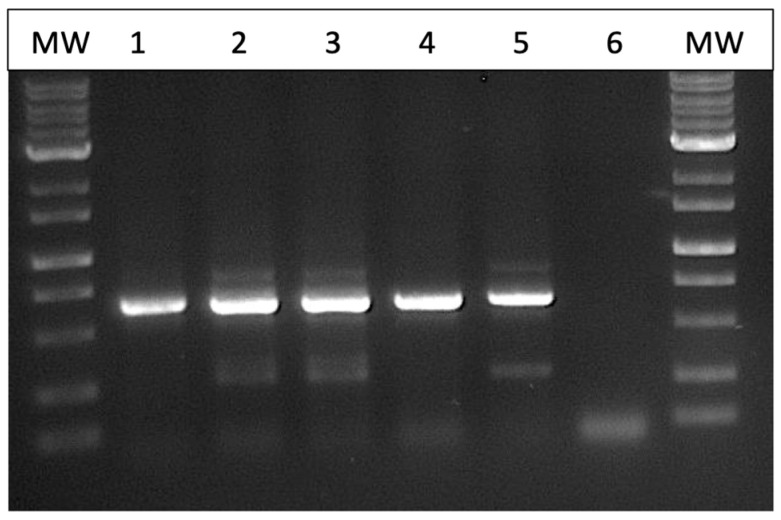
Electrophoresis gel following PCR using the EF-1a primer set of fungal colonies recovered from cannabis and tall fescue inflorescences. MW = molecular-weight ladder; lane 1 = positive control of *Trichoderma harzianum*; lanes 2 and 3 = *Fusarium avenaceum* from tall fescue plants; lane 4 = *Fusarium graminearum* from cannabis plants; lane 5 = *Fusarium avenaceum* from cannabis plants; lane 6 = control (no DNA).

*Fungal isolation from plants and identification.* Fungal colonies recovered from dried cannabis inflorescences and sub-cultured onto PDA+S were represented by two major morphological types. The first were slow-growing colonies with irregular margins and cottony aerial mycelia, and the underside of the colonies were beige with a tint of red (Figure 2a,b). The second type was represented by fast-growing red-pigmented colonies with some aerial mycelia (Figure 2c,d). Many fungal colonies recovered from tall fescue plants, when sub-cultured to fresh medium, resembled these two colony types (Figure 2e,f), in addition to other colonies that were either pigmented red or black, or were not pigmented. The tall fescue plants sampled and the colonies recovered from them are shown in Figure 3. Among the six isolates of *Fusarium* spp. that were subjected to PCR of the ITS1-ITS4 region of rDNA and of the EF-1α region, two species were identified—*F. avenaceum* and *F. graminearum* (Figure 4). Both species were recovered from cannabis and tall fescue inflorescences, with homologies in the range of 99.4–99.6% for both gene regions according to BLAST analysis (https://blast.ncbi.nlm.nih.gov/Blast.cgi, accessed on 4 September 2024). Three additional cultures that were pigmented red or black were identified as *Epicoccum nigrum*, *Sordaria macrospora*, and *Alternaria alternata*, with 100%, 99.6%, and 99.4% homology, respectively, to the ITS region, and all of them were recovered from tall fescue leaves.

**Figure 5:**
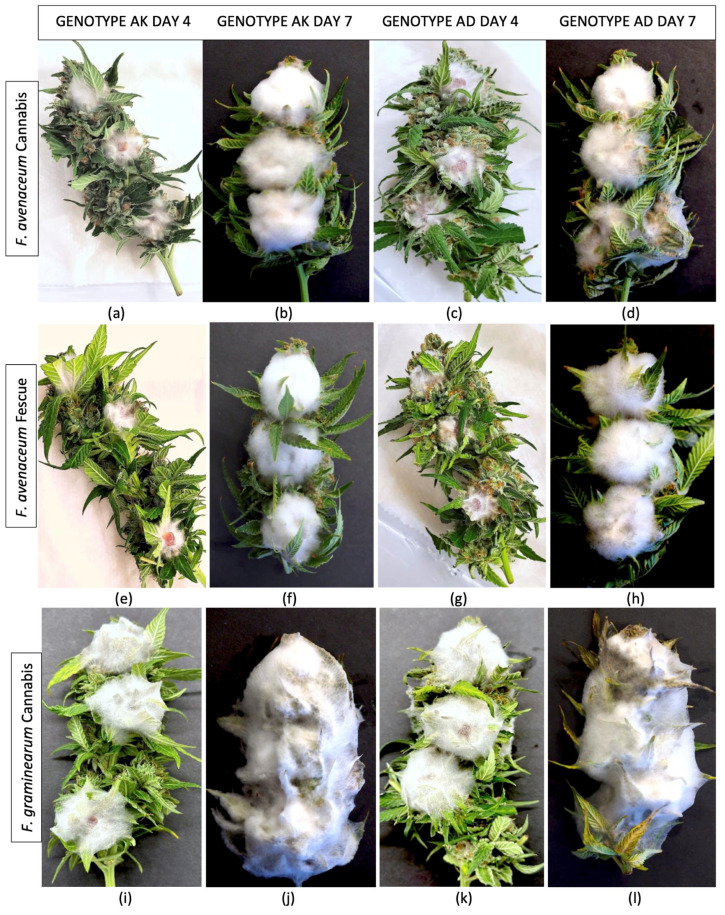
Mycelial colonization of detached cannabis inflorescences following inoculation with mycelial plugs of *Fusarium* spp. Two cannabis genotypes were included and photos were taken after 4 and 7 days of incubation under high humidity conditions in the laboratory. (**a**–**d**) Genotypes AK and AD inoculated with *Fusarium avenaceum* from cannabis. (**e**–**h**) Genotypes AK and AD inoculated with *Fusarium avenaceum* from tall fescue. (**i**–**l**) Genotypes AK and AD inoculated with *Fusarium graminearum* from cannabis.

**Figure 6:**
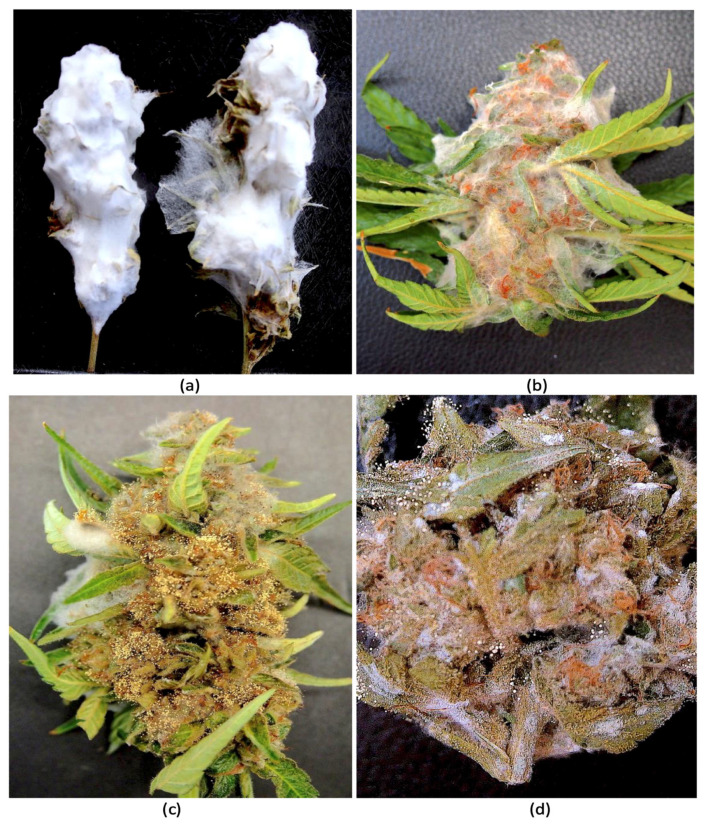
Colonization of detached cannabis inflorescences following inoculation with various fungi and incubation for 7 days. (**a**) Inoculation with *Fusarium graminearum* followed by drying of the tissues. (**b**) Inoculation with *Fusarium sporotrichiodes.* (**c**) Inoculation with *Aspergillus ochraceus*. (**d**) Inoculation with *Penicillium citrinum*.

*Pathogenicity tests.* Following the inoculation of freshly harvested detached cannabis inflorescences with mycelial plugs of *F. avenaceum* and *F. graminearum*, extensive mycelial growth was observed after 4 and 7 days on each of the two cannabis genotypes (AK, AD) due to the high humidity conditions within the sealed plastic containers (Figure 5). The *F. avenaceum* isolates from cannabis and tall fescue plants (FB1, FB2, FB3) grew similarly and there were no observable differences in the extent of mycelial colonization (Figure 5a–h). After 7 days, a ball of mycelium formed at each inoculation site. In contrast, inoculation with *F. graminearum* resulted in the complete colonization of the inflorescences after 7 days, resulting in a mummified appearance (Figure 5i–l). When these samples were dried, they retained the physical appearance of a mummified structure (Figure 6a). Inflorescences inoculated with *F. sporotrichiodes* were also covered by mycelial growth after 7 days, but to a much lesser extent compared to *F. graminearum* (Figure 6b). Inoculations with *A. ochraceus* and *P. citrinum* resulted in considerable sporulation by these species as shown in Figure 6c,d. These samples were similarly dried and used for mycotoxin analysis.

*Mycotoxin occurrence.* From the 8 samples of cannabis tissues that were naturally infected or artificially infected by 3 *Fusarium* species on 2 cannabis genotypes, a total of 14 mycotoxins were detected as shown in Table 2. From the naturally infected, dried cannabis inflorescences (buds) that were confirmed to contain *F. avenaceum* by isolation in pure culture and PCR identification, the mycotoxins BEAU and enniatins A1, B, and B1 were present at concentrations ranging from 0.063 to 1.78 μg/g. In cannabis inflorescences inoculated with an isolate of *F. graminearum* recovered from cannabis plants, the mycotoxins 3ADON and DON accumulated at levels of 0.34 and 1.91 μg/g, respectively, on cannabis genotype AK. In addition, ZEAR was present at a level of 56.2 μg/g (Table 2).

On cannabis genotype AD, these mycotoxin levels were 0.13, 1.18, and 31.8 μg/g, which were much lower than in genotype AK under the same conditions. In addition, the mycotoxin culmorin was present at a level of 0.38 μg/g in genotype AD.

In cannabis inflorescences inoculated with *F. avenaceum* originating from cannabis, the mycotoxins enniatins A, A1, B, and B1 were produced at varying levels. These levels were lower in tissues of genotype AD compared to AK, as observed previously with the mycotoxins 3ADON, DON, and ZEAR. In addition, BEAU was produced on genotype AD. In cannabis inflorescences inoculated with *F. avenaceum* originating from tall fescue spikes, the mycotoxins enniatins A, A1, B, and B1 were produced at varying levels, with higher levels detected in genotype AK compared to AD as before. BEAU was present in both genotypes, with higher levels in genotype AD. Lastly, in *F. sporotrichiodes*-inoculated tissues, the mycotoxins HT2 and T2 accumulated at levels of 13.9 and 10.9 μg/g, respectively. In addition, BEAU was also present at 0.133 μg/g (Table 2).

There were some unexpected results regarding the mycotoxins that were detected. For example, the mycotoxin alternariol was present in tissues of cannabis genotype AK inoculated with *F. graminearum* (FG) and *F. avenaceum* FB3 from tall fescue. As well, the mycotoxin tentoxin was present in tissues of genotypes AK and AD inoculated with *F. avenaceum* from tall fescue. In addition, the mycotoxins 3ADON and DON were present in tissues of both cannabis genotypes inoculated with *F. avenaceum* isolates from cannabis (FB1, FB2) and tall fescue (FB3). Control (uninoculated) cannabis tissues were devoid of mycotoxins, except for culmorin that was present at 0.38 μg/g. Inflorescences inoculated with *A. ochraceus* contained no mycotoxins with those receiving *P. citrinum* containing 6.1 μg/g of BEAU.

To explain the possible causes of the unexpected results above, tissues from which the mycotoxins were obtained were subsequently used for DNA extraction and were subjected to RT-qPCR using species-specific primers for *F. graminearum* and *F. avenaceum* [10]. In tissues inoculated with these two species, the corresponding DNA sequences were present (Table 2). However, in tissues that were inoculated with *F. avenaceum* only, the presence of *F. graminearum* was confirmed, suggesting some background infection had potentially occurred from natural inoculum at the time the inflorescences were harvested for use in this study. These findings can explain the spurious presence of mycotoxins that were likely to be associated with incipient infections by *F. graminearum* in samples where the tissues were either not inoculated or were inoculated with a different species.

## 4. Discussion

*Fusarium* species are widespread pathogens of crop plants and cause a broad range of disease symptoms. Up to 16 species of *Fusarium* have been reported to be associated with cannabis plants and are classified in 6 species complexes: *Fusarium oxysporum, F. solani*, *F. incarnatum-equiseti, F. sambucinum, F. tricinctum*, and *F. fujikuroi*. Among these, a sub-set of the species that cause diseases on cereal crops are known to produce a range of mycotoxins that can cause problems following ingestion by humans and animals [14,15]. In particular, the *Fusarium* species causing head blight or scab symptoms on wheat are the most well characterized with regard to the range of mycotoxins produced [14,15,16]. On cannabis plants, infection by two cereal-infecting *Fusarium* species, namely *F. graminearum* and *F. sporotrichiodes*, has been previously reported [1,2,9]. However, the potential of these species to produce mycotoxins in cannabis tissues has not been previously investigated.

In the present study, isolates of *F. graminearum* and *F. sporotrichioides* and a species previously unreported from cannabis, *F. avenaceum,* all isolated from symptomatic fresh and dried inflorescences, were tested for mycotoxin production under controlled conditions. It should be noted that the inoculated cannabis tissues were incubated under high relative humidity (>90%) for a prolonged period (7 days) prior to mycotoxin analyses being conducted. Similarly, asymptomatic control tissues were exposed to these incubation conditions. Where present, these conditions would have allowed undetected incipient infections to progress before the assays were performed. Under normal greenhouse-growing conditions, these environmental conditions would not be expected to occur unless air circulation was compromised and temperature and relative humidity levels exceeded the normal range (55% relative humidity and temperatures in the range of 27–30 °C). Such conducive conditions (70% relative humidity and temperatures in the range of 34–36 °C) can occur in greenhouses at certain times of the year, resulting in outbreaks of diseases, such as Botrytis bud rot, caused by *B. cinerea*. This disease is favored by humid conditions that occur from July to October, resulting in disease outbreaks [17]. In the present study, the development of pinkish-white mycelium of *Fusarium* was observed on some plants growing under greenhouse conditions in late summer, suggesting the environmental conditions were conducive for infection to occur at these time periods. In addition, the relative humidity and temperature within inflorescences are reported to be higher than the ambient environment [10], indicating that microclimatic conditions may be conducive for infection by *Fusarium* spp. as they are for *B. cinerea* [17]. Such conditions can result in the development of *F. avenaceum* and *F. graminearum* in inflorescence tissues prior to packaging, a situation also similarly encountered with *B. cinerea* infections [17]. The incidence of visible infections due to *Fusarium*, however, appears to be less common on greenhouse-grown cannabis plants when contrasted with outdoor-grown hemp plants. Outdoors, *Fusarium* spp. were reported to infect inflorescences and produce mycotoxins, and prolonged periods of rain and high humidity (75–80%) were associated with outbreaks of disease [6,7,8]. The incidence of detection of various *Fusarium* spp. within these samples was 95%, indicating that the potential for contamination by mycotoxins in outdoor-grown hemp is significantly higher than for indoor-grown cannabis. In addition, the post-harvest drying and storage conditions for hemp can lead to the extensive growth of *Fusarium* spp. [6,8]. While the sources of inoculum for hemp may include spores from adjacent fields planted to cereal crops, the inoculum sources for indoor-grown cannabis have not been previously identified.

In the present study, tall fescue plants that were grown as a part of the urban landscape within a 30 m distance from the greenhouse were observed to harbor both *F. avenaceum* and *F. graminearum*. Isolates of both species recovered from spikelet tissues of this host were capable of infecting cannabis inflorescences under laboratory conditions and produced mycotoxins in these tissues. The mycotoxins accumulated were reflective of the infecting species, with some exceptions, and were produced at significant levels (Table 2). The importance of wild grass species, including tall fescue plants, growing adjacent to wheat fields in providing a source of inoculum for initiation of disease has been confirmed in several previous studies. At a field site located in Germany, Gerling et al. [18] reported that grasses belonging to *Lolium* spp. growing next to wheat production fields impacted the *Fusarium* species composition and mycotoxin accumulation in wheat plants, particularly those located adjacent to the grasses. The most prevalent species of *Fusarium* recovered from the grasses, and shown to be pathogenic on wheat plants, were *F. graminearum*, *F. culmorum*, and *F. sporotrichioides*. A low level of recovery of *F. avenaceum* was also reported. Wheat kernels produced on plants located next to the infected grasses had higher *Fusarium* abundance and mycotoxin accumulation compared to those located at further distances [18]. The use of irrigation enhanced the abundance of *Fusarium* spp. on both the grasses and on the wheat plants when compared to non-irrigated sides of the field, with *F. graminearum* being recovered at the highest frequency. Sampling at various distances from the grasses revealed a gradient in percent recovery from the wheat plants, with the highest recovery observed at a 1 m distance and lowest at 33 m. These results demonstrate that the grasses can be a reservoir of inoculum for *Fusarium* spp. that subsequently spread into the wheat field [18].

Similar findings were obtained from field studies conducted in New York State, where *F. graminearum* was shown to be prevalent within spikes of various wild grass species, including tall fescue, especially in irrigated sites adjacent to agricultural fields [19]. *Fusarium avenaceum* was also recovered from a number of different grass species [20] and it has been shown to be seed-borne in tall fescue production sites in Oregon [21], and was shown to infect tall fescue and orchard grass plants grown for pasture [22]. These observations support the hypothesis that *Fusarium* inoculum produced in infected spikes on tall fescue plants growing adjacent to a cannabis greenhouse facility could potentially have spread and infected cannabis inflorescences within the greenhouse. While both *F. graminaearum* and *F. sporotrichioides* have been previously recovered from diseased cannabis and hemp inflorescences [5,6,9,10], they are not reported to infect other parts of the cannabis plant, such as roots and stems, and hence do not provide a potential source of inoculum. Since there are strict biosecurity protocols in place for indoor production of cannabis to meet regulatory guidelines [17], other inoculum sources of these cereal-infecting species, such as worker’s clothing or boots, are unlikely. Air-borne inoculum from external sources is the most likely source, which can enter through open greenhouse vents.

In addition to the mycotoxins associated with the three *Fusarium* spp. included in this study, other mycotoxins were detected in dried cannabis inflorescences, such as alternariol and tentoxin, which were present in three samples. The likely source of these mycotoxins is from *Alternaria* spp., which are known to infect cannabis plants [23]; they are also commonly present in grain samples similarly colonized by this pathogen [13,24]. While the inflorescence samples collected for artificial inoculation studies did not show external symptoms due to any pathogen, pre-existing latent infections were likely to have been present that continued to progress during the high relative humidity conditions under which the tissues were incubated. Similar *Alternaria* toxins were also reported to be present at high concentrations in dried hop flowers [25] and in hemp seeds [26]. In hop flowers, DON accumulation from *Fusarium* spp. was detected in all the samples tested. Mycotoxins normally associated with *Aspergillus* or *Penicillium* species colonizing plant tissues, including aflatoxins and ochratoxins, were not detected in hop flowers. They were also absent in a large-scale sampling of dried cannabis flowers conducted in California [27].

In the present study, artificial inoculations conducted with isolates of *A. ochraceus* and *P. citrinum* derived from cannabis inflorescences did not give rise to any of the ochratoxins normally produced by these species [16,28] in the inoculated tissues, although additional studies may be needed to determine the mycotoxin potential of these species in cannabis. Other aflatoxin-producing species of *Aspergillus* were not included in this study as they were unavailable at the time this study was initiated. Currently, there is not a strong body of evidence to support that aflatoxins and ochratoxins are prevalent in cannabis samples originating from production sites that follow regulatory guidelines during production. However, these mycotoxins have attracted attention from regulatory agencies as their known carcinogenic properties can present potential risks to immunocompromised humans if exposed to levels that exceed certain thresholds [3]. There is a growing body of evidence, including data from the current study, which indicates that *Fusarium* and *Alternaria* toxins may actually be more ubiquitous in cannabis tissues. In future studies, these mycotoxins should receive more attention to determine their presence and if the establishment of threshold limits is required and if monitoring for their presence in cannabis tissues should be enforced.

Incipient infections by *F. graminearum* were confirmed to be present in asymptomatic fresh inflorescences samples used for inoculation with *F. avenaceum* by RT-qPCR. Pathogen presence explains the unexpected detection of mycotoxins associated with this species. It should be noted that these tissues were tested for mycotoxin presence after 7 days of incubation under high relative humidity conditions. By this time, mycelial growth had encompassed the cannabis tissues, a situation not commonly observed in commercial growing environments. It is not certain whether natural infections would have proceeded to develop and accumulate mycotoxins under normal cultivation practices. In the absence of a known source of *Fusarium* inoculum, such as the tall fescue plants identified in the present study, the risk levels for mycotoxin accumulation would appear to be low. While *Fusarium* mycotoxins and various fungal metabolites were reported to be present in seized samples from illicit growing operations in Arizona and California [4], the growing environments depicted do not reflect the production environments encountered in legalized production sites.

In the future, a large-scale systematic study of cannabis inflorescences for the presence of *Fusarium* mycotoxins in licensed production facilities should shed light on the extent to which they accumulate in inflorescence tissues produced under greenhouse conditions. It also remains to be determined what levels of relative humidity conditions could promote the development of *Aspergillus* and *Penicillium* species to the level of colonization that would be associated with ochratoxin and aflatoxin accumulation. Based on the observations from the present study, the risks of contamination with these toxins remain low within licensed production facilities. The current findings, also supported by the recent published literature, suggest that the potential for *Alternaria* and *Fusarium* colonization of cannabis and hop tissues is more likely to lead to mycotoxin presence, and this could represent a moderate risk level. Some of these toxins—enniatins, BEAU, and *Alternaria* toxins—can be classified as “emerging mycotoxins”, defined as “mycotoxins which are neither routinely determined nor legislatively regulated”. However, the evidence of their incidence is rapidly increasing according to Chiotta et al. [16]. Future studies should target these groups of mycotoxins to determine whether or not the current regulations surrounding mycotoxins in cannabis should be modified to include them.

The two cannabis genotypes included in this study that were artificially inoculated produced comparable rates of tissue colonization by mycelium but differed in the levels of mycotoxins that were present, in some cases differing by as much as four-fold. These differences could be attributed to the different genetic backgrounds of these genotypes (Supplementary Figure S1), but the mechanism remains unexplained. However, they are highly interesting as they suggest there may be differences in the extent to which certain genotypes of cannabis may accumulate these mycotoxins, with some apparently showing lower levels. In wheat samples, differences in the accumulation of mycotoxins in harvested grains were reported to be influenced by the cultivar, fungal isolate, and geographic location, suggesting that the complex interactions between the pathogens, different cultivars, and the environment are important in determining disease outcomes and mycotoxin accumulation [13].

In summary, this study indicates the potential for disease development and mycotoxin accumulation in cannabis inflorescence tissues following infection by *Fusarium* species that commonly infect graminaceous hosts, including cereal crops and other grasses. In view of the findings from this study, there may be a need to develop best practices that limit the spread of inoculum and introduction of *Fusarium* species from neighboring alternate hosts. These may include the elimination of these hosts if they are adjacent to a greenhouse facility to provide a clear buffer zone, as well as spore-monitoring studies to confirm inoculum presence. In addition, research is needed to better understand the interactions between cannabis cultivars and *Fusarium* fungal isolates, since there are known to be differences in cultivar responses to mycotoxin accumulation in cereal grains [29,30,31]. Furthermore, studies on the environmental conditions leading to disease development and mycotoxin accumulation in cannabis are needed, both in greenhouse-grown and outdoor-grown crops, since these factors can influence mycotoxin accumulation in cereal crops [29,32]. Lastly, targeted disease management approaches and assessing the potential for breeding for resistance to infection by *Fusarium* species and mycotoxin accumulation in cannabis and hemp should be explored to better manage this unique host–pathogen interaction.

## Figures and Tables

**Table 1:** Mycotoxin analytes and limits of quantitation (LOQs).

Mycotoxin	Abbreviation	LOQ (ug/kg)
Deoxynivalenol	DON	50
Deoxynivalenol-3-glucoside	D3G	50
3-acetyl deoxynivalenol	3ADON	50
15-acetyl deoxynivalenol	15ADON	50
Culmorin	CUL	20
Nivalenol	NIV	50
7-α hydroxy,15-deacetylcalonectrin	3ANX	80
7-α hydroxy, 3,15-dideacetylcalonectrin	NX	100
Zearalenone	ZEAR	24
Fumonisin B1	FB_1_	24
Fumonisin B2	FB_2_	24
Beauvericin	BEAU	2
Enniatin A	ENN A	2
Enniatin A1	ENN A1	5
Enniatin B	ENN B	20
Enniatin B1	ENN B1	16
HT-2 toxin	HT2	30
T-2 toxin	T2	20
Tentoxin	TENT	4
Alternariol methyl ether	AME	4
Altenuene	ALT	20
Alternariol	AOH	6
Aflatoxin B_1_	AFB_1_	1
Aflatoxin B_2_	AFB_2_	1
Aflatoxin G_1_	AFG_1_	1
Aflatoxin G_2_	AFG_2_	1
Ochratoxin A	OTA	1
Citrinin	CIT	3

**Table 2:** Response of two genotypes of cannabis (AD, AK) to inoculation with three species of Fusarium isolated from two hosts (cannabis, fescue) with regard to mycotoxin accumulation in inflorescence tissues compared to naturally infected tissues. Mycotoxin levels were determined using an HPLC-MS/MS method.

	Naturally Infected Bud Tissues (*F. avenaceum*)	*Fusarium graminear.* (Cannabis)	*Fusarium graminear*. (Cannabis)	*Fusarium avenaceum* (Cannabis)	*Fusarium avenaceum* (Cannabis)	*Fusarium avenaceum* (Fescue)	*Fusarium avenaceum* (Fescue)	*Fusarium sporotrich*. (Cannabis)
**CANNABIS GENOTYPE**	AD	AK	AD	AK	AD	AK	AD	AK
**MYCOTOXINS DETECTED (μg/g)**								
3-acetyl DON	-	0.34	0.13	-	-	0.073 *	-	-
Alternairol	-	0.159	-	-	-	0.151	-	-
DON	-	1.91 *	1.18 *	0.142 *	0.03 *	0.51 *	0.08 *	-
Zearalenone	-	56.2	31.8	-	-	-	-	-
Beauvericin	0.098	-	-	-	2.43	0.008	0.051	0.133
Culmorin	-	-	0.38 *	-	-	-	-	-
Enniatin A	-	-	-	0.028	0.019	0.042	0.011	-
Enniatin A1	0.063	-	-	0.621	0.358	0.714	0.181	-
Enniatin B	1.78	-	-	9.3	6.7	7.67	3.96	-
Enniatin B1	0.395	-	-	5.89	2.95	4.37	1.40	-
HT2	-	-	-	-	-	-	-	13.9
T2	-	-	-	-	-	-	-	10.9
Tentoxin	-	-	-	-	-	0.033	0.049	-

Control uninoculated cannabis inflorescences used in this study were found to be free of mycotoxins except for the detection of culmorin at 0.38 μg/g. * The presence of DNA of *F. graminearum* was confirmed in these samples by RT-qPCR using the methods described by Bamforth et al. [10]. The color coding reflects the characteristic mycotoxins produced by the representative *Fusarium* or *Alternaria* species. Light blue = *F. graminearum*; green = *F. avenaceum* and *F. sporotrichiodes*; yellow = *F. avenaceum* and *F. sporotrichiodes*; purple = *F. sporotrichiodes*; red = *Alternaria* species.

## Data Availability

The original contributions presented in this study are included in the article; further inquiries can be directed to the corresponding author.

## Supplementary Materials

The following supporting information can be downloaded at: https://www.mdpi.com/article/10.3390/jof11070528/s1, Figure S1: Characteristics of cannabis genotypes AK and AD used for inoculation with *Fusarium* species and mycotoxin analysis in this study.
