# Supplementary material for: Fusarium Species Infecting Greenhouse-Grown Cannabis (Cannabis sativa) Plants Show Potential for Mycotoxin Production in Inoculated Inflorescences and from Natural Inoculum Sources
Source: J Fungi (Basel). 2025 Jul 16;11(7):528. doi: 10.3390/jof11070528 (PMC12296103; doi:10.3390/jof11070528)
Supplement: Supplementary file 1 [file jof-11-00528-s001.zip › jof-3678771-supplementary.pdf]

Phenotypic trait	Genotype AK	Genotype AD
Total yeast and mold levels	Moderate	High
Botrytis cinerea suceptibility	High (>5% infection)	High (>5% infection)
Powdery mildew suceptibility	High	High
THC range	19-25%	20-28%
CBD range	0-1 %	0-1%
Top five terpenes (Highest to Lowest)	Caryophyllene<br>Bisabolol<br>Humulene<br>Linalool<br>Myrcene	Caryophyllene<br>Myrcene<br>Limonene<br>Linalool<br>Humulene
Genotype characteristic	Indica	Hybrid

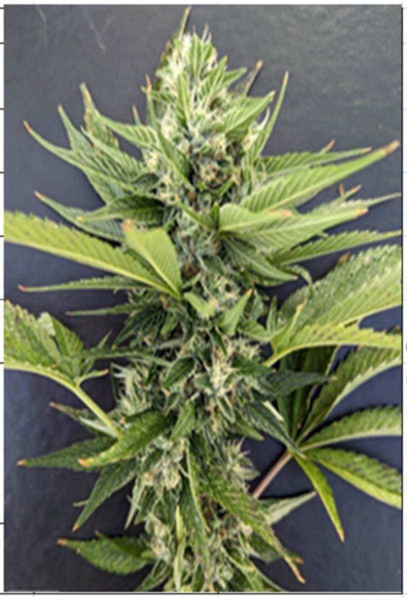

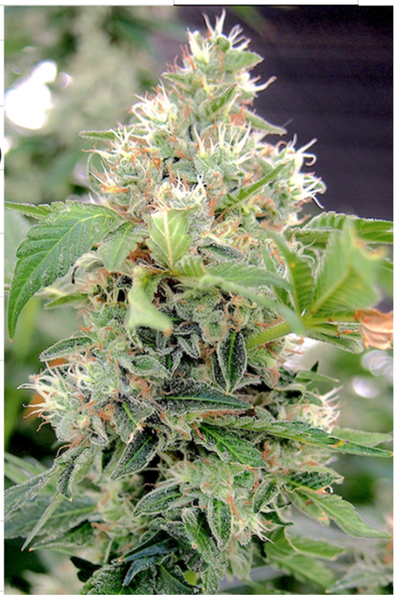

Supplementary Figure S1. Characteristics of cannabis genotypes AK and AD used for inoculation with *Fusarium* species and mycotoxin analysis in this study.
